# Resistance Exercise in Treating Heart Failure with Preserved Ejection Fraction (HFpEF) and Obesity: Targeting Skeletal Muscle Abnormalities and Ectopic Adipose Depots

**DOI:** 10.3390/physiologia5010010

**Published:** 2025-02-27

**Authors:** Daniel J. McDonough

**Affiliations:** 1 Division of Epidemiology and Community Health, School of Public Health, University of Minnesota-Twin Cities, Minneapolis, MN 55455, USA;; 2 K99/R00 Postdoctoral Research Fellowship Program, National Heart, Lung and Blood Institute, National Institutes of Health, Bethesda, MD 20892, USA

## 1. Introduction

Heart failure is a leading cause of morbidity and mortality worldwide [1], and with continued increases in the global prevalence of key disease drivers like physical inactivity and obesity in an aging population [2–7], heart failure with preserved ejection fraction (HFpEF; a left ventricular ejection fraction ≥50%) is now the dominant heart failure sub-type [8–10]. Accordingly, HFpEF is one of the most urgent prevention and treatment challenges in public health today given its increasing prevalence, limited therapeutic options, and the substantial burden on global health care systems [8,9]. The majority of HFpEF patients have obesity (a body mass index ≥30 kg/m^2^) [11–15], and growing evidence suggests that obesity and excess adiposity are not merely comorbidities, but may play a central role in the pathogenesis and progression of HFpEF [12,13,16–18]. HFpEF with obesity is pathophysiologically distinct from HFpEF without obesity and is characterized by more adverse hemodynamics, higher risk of heart failure hospitalization, worse symptomatology (e.g., dyspnea), and more severe exercise intolerance—the primary hallmark of chronic HFpEF [11,12,15,19–22].

The obesity-driven genesis of regional ectopic adipose depots, independent of total adiposity, may particularly exacerbate exercise intolerance in HFpEF and worsen its prognosis through auto-, para-, and endocrine pathways [11,13]. Ectopic visceral adipose tissue (VAT) in the intra-abdominal and thoracic (i.e., epicardial, pericardial, and perivascular) compartments is a metabolically active secretome, which acts as an independent organ, producing, expressing, and secreting exosomes and adipokines that are proinflammatory, oxidative, vasoactive, and angiogenic [13,18,23–28]. These harmful substances exert negative downstream effects on systemic inflammation, insulin sensitivity, lipid regulation, and blood pressure [11,13,18,29–31]. Furthermore, the ectopic accumulation of intra-hepatic triglycerides (hepatic steatosis) produces, expresses, and secretes proinflammatory hepatokines and increases resistance in the hepatic sinusoids, which disrupts transhepatic blood flow to the heart and limits preload reserve, further exacerbating exercise intolerance in HFpEF [9,30–40]. In skeletal muscle, the ectopic accumulation of intra- and inter-myocellular triglycerides (myosteatosis) further contributes to exercise intolerance in HFpEF via skeletal muscle function deficit, impaired tissue perfusion (oxygen delivery), and oxygen utilization (aerobic metabolism) [13,41–44].

Exercise intolerance in HFpEF also manifests from other skeletal muscle abnormalities, including sarcopenia, lower type I (oxidative) muscle fibers and type I-to-type II fiber ratio, capillary rarefaction and reduced capillary-to-fiber ratio, and reduced mitochondrial number and size and mitochondrial dysfunction [41–43,45,46]. Thus, given the terminal differentiation and limited rejuvenation capacity of the heart in HFpEF, and plasticity and rejuvenation capacities of adipose and skeletal muscle tissues, there have been recent calls for therapies targeting extracardiac, cardiometabolic perturbations in the treatment of HFpEF [9,41–43,45,46].

## 2. Pharmacotherapy and Aerobic Exercise-Based Treatments in HFpEF

Historically, pharmacological trials in HFpEF have not shown benefit for improving exercise capacity (measured as peak oxygen consumption) or quality of life (QoL) in HFpEF [47–51]. However, recent trials of pharmacotherapy with anti-obesity medications (e.g., semaglutide, tirzepatide) have shown promise for improving peak oxygen consumption and QoL in patients with HFpEF and obesity [52,53]. However, these medications are expensive with financial and social barriers to access [54–59]; discontinuation rates are high and reinitiation rates are low with a high likelihood of weight (fat) regain following cessation [60]; there is a risk of gastrointestinal adverse events [61]; and drug-induced weight loss has been observed to result in significant loss of lean body mass [62–66], which may compromise long-term functional capacity and independence in an already frail patient population [41–43,45,46,67,68].

Exercise trials in HFpEF have also demonstrated clinically meaningful improvements in peak oxygen consumption and QoL [69–81]. However, exercise trials in HFpEF have been primarily aerobic-based and have had poor adherence (~50–70% complete 100% of exercise sessions) [75,80–84]. Traditional aerobic exercise (e.g., cycle ergometry) requires continuous movement of multiple, large leg muscles (>6 kg of muscle mass) and is, therefore, limited by the heart in HFpEF [85], which is particularly troubling for HFpEF patients with obesity who suffer from worse aerobic capacity and more severe exercise intolerance [11,12,15,19–22,43,76,86–90]. A meta-analysis [91] found resistance exercise, alone, vs. concurrent exercise training (aerobic + resistance), similarly improves peak oxygen consumption and QoL in patients with HF with *reduced* ejection fraction (HF*r*EF) [91]. Thus, considering that HFpEF patients are generally older, more frail, and have greater functional limitations than HF*r*EF patients [67,68,89], resistance exercise, alone, may be sufficient to target the cardiac and extracardiac contributors of exercise intolerance in patients with HFpEF and obesity [89,91,92]. However, there appears to be no trial to date isolating the effect of resistance exercise in this population [82,83,89].

## 3. Potential of Resistance Exercise in Treating HFpEF

HFpEF is a full-body disorder and, thus, may benefit most from full-body therapies. Resistance exercise, alone, can simultaneously reduce VAT [93–96], myocardial steatosis [97–99], hepatic steatosis [100–103], and myosteatosis [104,105], without diet-induced weight loss [106,107], while requiring less [objectively measured] exertional effort than aerobic exercise [108,109]. Unlike aerobic exercise, selectively targeting smaller muscle masses (‘small muscle exercise’), as with full body resistance exercise, is not limited by the heart, such that individual muscles can be trained, followed by periods of rest, before being repeated [85]. Furthermore, resistance (but not aerobic) exercise can selectively target all major skeletal muscles and concomitantly improve the skeletal muscle-specific contributors to exercise intolerance in HFpEF and obesity on a whole-body level (discussed below). In particular, low-load/high-repetition resistance exercise (exercising at ~30% of patients’ 1-repetition maximum [1RM] on a given exercise) has been recommended by the American Heart Association [110] as an alternative to aerobic exercise in cardiovascular disease patients with compromised aerobic capacity, and has been deemed safe and suitable for frail elderly people given its low articular stress and low risk of cardiac events compared to traditional resistance exercise at moderate and high loads [110–121].

**Figure 1.**
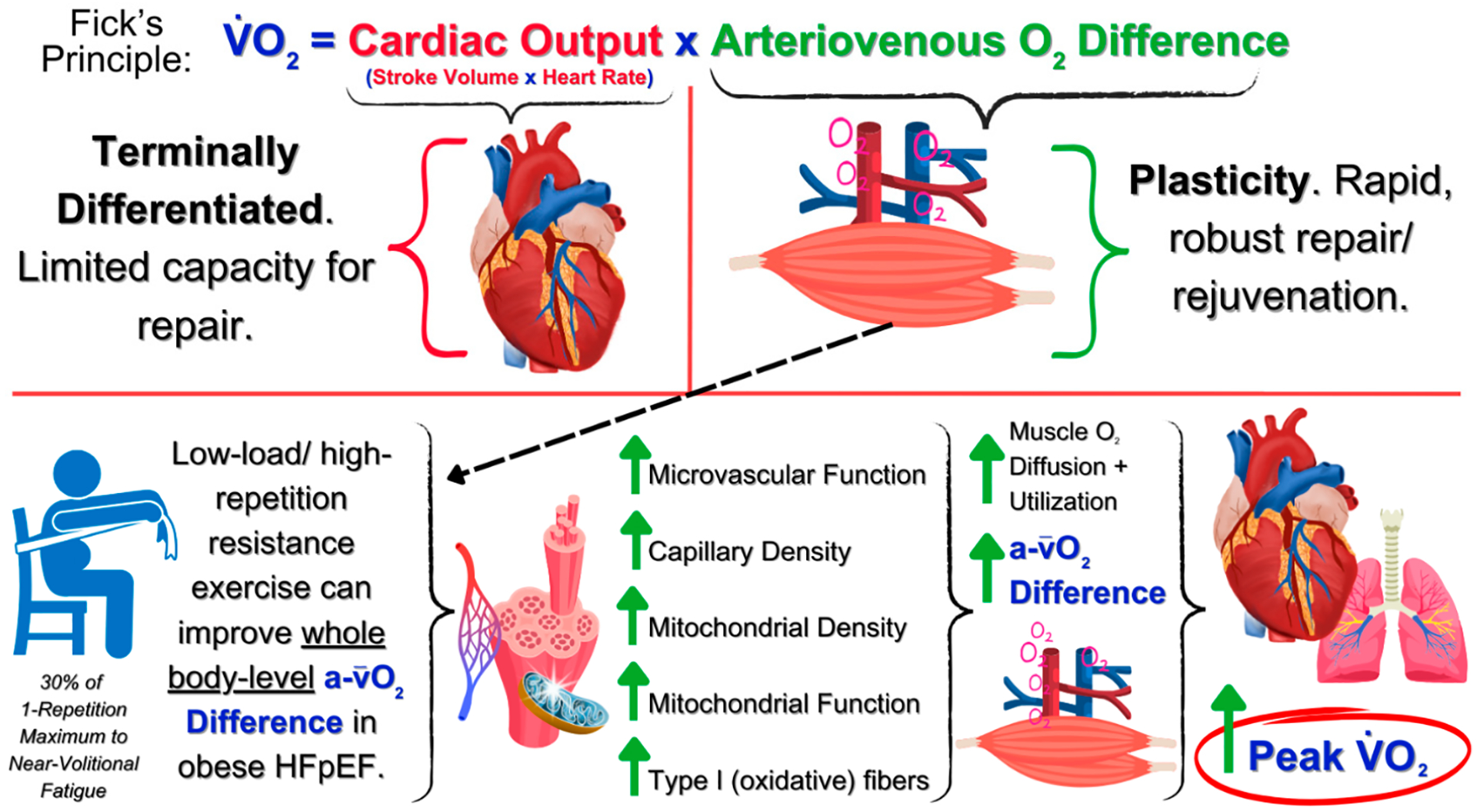
Summary of the potential for low-load/high-repetition resistance exercise to satisfy Fick’s Principle (equation) by targeting the peripheral (non-cardiac), skeletal muscle-specific contributors of exercise intolerance in patients with HFpEF and obesity to improve exercise capacity (peak oxygen consumption). Abbreviations: O_2_ = oxygen; $\overset{\cdot }{\text{V}}{\text{O}}_{2}$ = maximal oxygen consumption; $\text{a}-\bar{\text{v}}{\text{O}}_{2}$ Difference = arteriovenous oxygen difference.

Low-load/high-repetition resistance exercise may be particularly effective for targeting the peripheral, skeletal-specific contributors of exercise intolerance in HFpEF and obesity (Figure 1) [41,43,122–125]. When performed to sub-volitional fatigue in older adults (i.e., a rating of perceived exertion [RPE] score of ~7 out of 10), low-load/high-repetition resistance exercise confers similar benefits in skeletal muscle morphology (e.g., hypertrophy) and function (e.g., muscular strength) as traditional, higher-load resistance exercise (≥70% 1RM) [111,115–120,126]. Furthermore, low-load/high-repetition resistance exercise mimics aerobic exercise in that energy utilization rapidly shifts to oxidative energy metabolism. Accordingly, chronic adaptations include transitioning type II (glycolytic) muscle fibers (highly prevalent in HFpEF and obesity) to type I (oxidative) fibers [127], improved mitochondrial content and bioenergetics, and oxygen perfusion and utilization on a whole-body level [116,128]. Likewise, low-load/high-repetition resistance exercise increases skeletal muscle capillarity and capillary-to-fiber ratio and improves microvascular function by elevating nitric oxide bioavailability in the vascular tissue—a therapeutic target in previous HFpEF trials [129–133]. Low-load/high-repetition resistance exercise may also help overcome perceived barriers to traditional resistance exercise in patients with HFpEF and obesity (e.g., articular stress and fear of pain, injury, and acute cardiac events) [111,134–136]. Thus, older adults who are allowed to self-select load in a resistance exercise program tend to choose lower loads (~20–30% 1RM) [137] rather than moderate or heavy loads and, thus, a low-load resistance exercise intervention may further promote long-term adherence to exercise therapies in patients with HFpEF and obesity. Finally, although there was initial reluctance in applying traditional resistance exercise at moderate and higher loads to patients with heart failure, low-load resistance exercise with resistance bands or weight-stack machines has been deemed safe [110,112–114,121,138].

## 4. Future Research Directions

Despite the potential of resistance exercise to improve exercise capacity and QoL in patients with HFpEF and obesity, clinical trials of resistance exercise, as a standalone intervention, have not yet been conducted. We have developed, and are currently initiating, a pilot randomized clinical trial funded by the National Heart, Lung and Blood Institute of the National Institutes of Health (HL173668) to examine the feasibility and acceptability of a full-body, low-load/high-repetition resistance exercise intervention, as a standalone intervention, in patients with HFpEF and obesity. Following this, we will develop and implement a multi-center randomized clinical trial examining the synergistic effects of low-load/high-repetition resistance exercise with pharmacotherapy-induced weight loss on all-cause hospitalization and mortality in patients with HFpEF and obesity [52,53,139,140]. Despite the above-noted limitations of pharmacotherapy with anti-obesity medications, their effects on key clinical outcomes in patients with HFpEF and obesity are remarkable. Adding resistance exercise may further enhance these effects by targeting problematic VAT compartments and other ectopic adipose depots while simultaneously *increasing* muscle mass, preserving or increasing bone density, improving skeletal muscle function on a whole-body level, and helping to prevent weight (fat) regain, further improving key therapeutic targets in this population [66,141]. Remote, home-based interventions in particular may better appeal to patients with HFpEF and obesity to minimize burden and maximize intervention adherence and retention [142,143].

**Figure 2.**
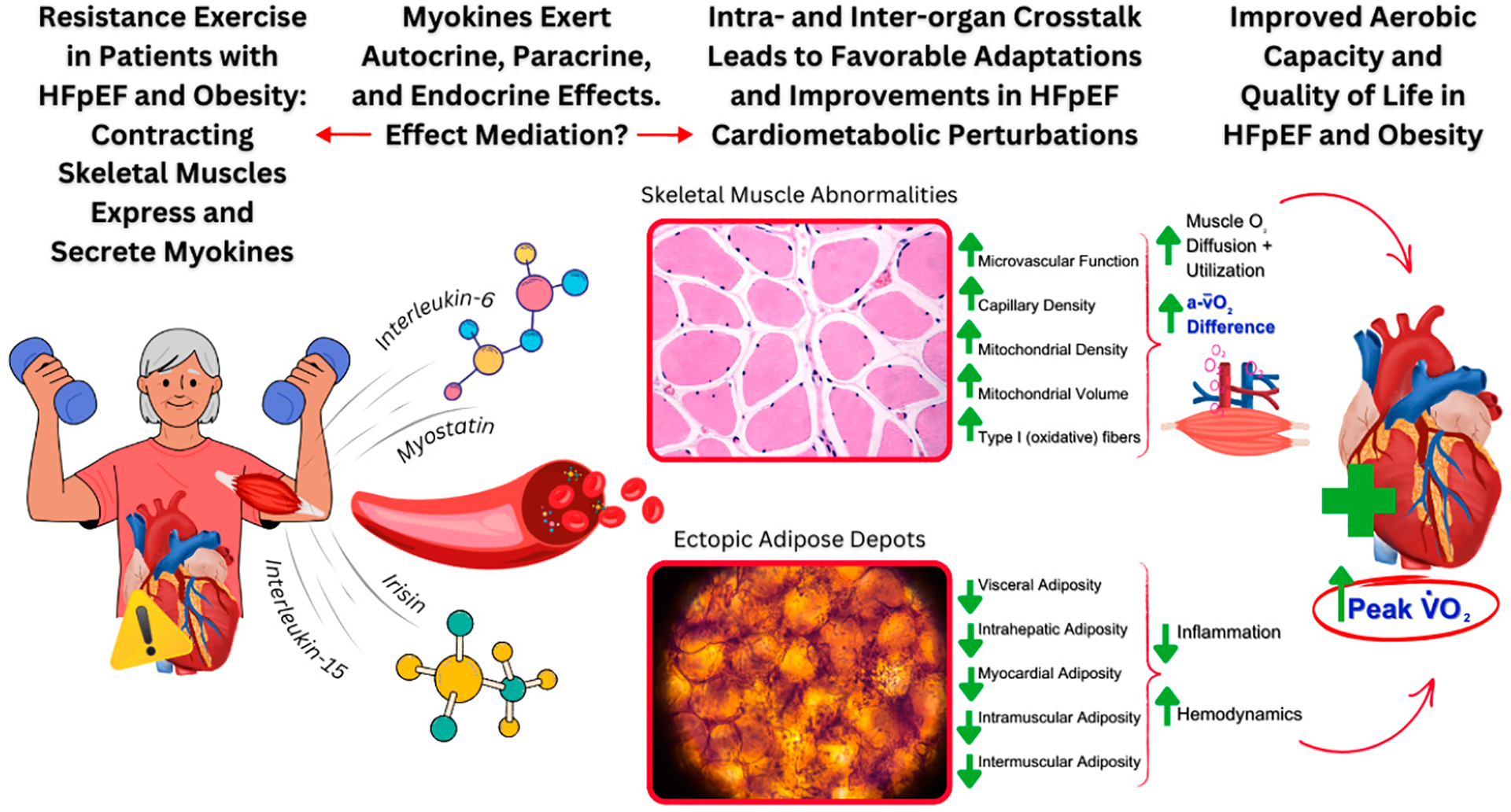
Summary of the potential (to be examined in future research) for low-load/high-repetition resistance exercise to improve the peripheral (non-cardiac), cardiometabolic perturbations (skeletal muscle abnormalities and ectopic adipose depots) in HFpEF and obesity, and the potential effect mediation of skeletal muscle-produced myokines that are secreted during muscular contractions. Abbreviations: Peak $\overset{\cdot }{\text{V}}{\text{O}}_{2}$ = peak oxygen consumption; $\text{a}-\bar{\text{v}}{\text{O}}_{2}$ Difference = arteriovenous oxygen difference.

Akin to adipose tissue, the skeletal muscle tissue secretome produces, expresses, and secretes myokines during skeletal muscle contraction (e.g., during resistance exercise), which exert beneficial auto-, para-, and endocrine effects through muscle–organ crosstalk, and may also interact with harmful adipokines in obesity and related diseases like HFpEF [144–154]. Research in this area is still in its infancy, but future research should examine their potential role in mediating the beneficial effects of resistance exercise in treating HFpEF and obesity (Figure 2).
